# *In vitro* fertilization: an unexpected finding in a cohort of patients with biliary atresia

**DOI:** 10.1590/1414-431X2023e12671

**Published:** 2023-03-24

**Authors:** C.M. Costa, A. Porta, I.K. Miura, G. Porta, E.A. Fonseca, R. Pugliese, M. Kondo, P. Chapchap, R. Sindhi, F.H. Feier, J. Seda

**Affiliations:** 1 Hepatologia e Transplante Hepático, Hospital Sírio-Libanês, São Paulo, SP, Brasil; 2 Hepatologia e Transplante Hepático, A.C. Camargo Cancer Center, São Paulo, SP, Brasil; 3 Hillman Center for Pediatric Transplantation, Thomas E. Starzl Transplantation Institute, Department of Transplant Surgery, University of Pittsburgh Medical Center Children's Hospital of Pittsburgh, Pittsburgh, PA, USA

**Keywords:** Portoenterostomy, Liver transplantation, *In vitro* fertilization, Children, Outcomes, Biliary atresia

## Abstract

In biliary atresia (BA), efforts to prevent premature liver transplantation (LT) are aimed at early diagnosis, timing of Kasai-portoenterostomy (KPE), and centralization of care. This report presents the clinical picture, treatment strategies, and outcomes of BA patients with no previous treatment. A retrospective cohort study (Jan/2001 to Jan/2021) was conducted to evaluate the outcome of patients with BA referred to a single team. Study groups were: 1) Kasai-only group (K-only) n=9), 2) LT-only group (n=7), and 3) Kasai+LT group (K+LT) (n=23). Survival with native liver and overall survival were 22.9 and 94.8%, respectively, at 120 months of follow-up. There was no difference in age at KPE in the K-only group (46.8±21.8 days) *vs* K+LT (52.1±22 days), P=0.4. Ten (25.6%) patients were babies conceived through *in vitro* fertilization (IVF). Four IVF patients (40%) presented associated congenital heart disease *vs* 5 patients (17%) in the remaining group (P=0.14). Two of the IVF patients were premature (<37 weeks). Median maternal age at birth was 35 years (33 to 41 years). Excellent patient survival is expected for patients with BA with the available treatment strategies. IVF+BA was an unexpected prevalent association in this cohort, and further studies are required to better understand these findings.

## Introduction

In patients with biliary atresia (BA), the efforts of preventing premature liver transplantation (LT) are aimed at early identification and diagnosis, timing of Kasai-portoenterostomy (KPE) before 45 days of age, and centralization of surgery at experienced centers (1-3). However, a recent analysis of patients with BA who underwent KPE in the United States showed that the majority were operated after 60 days of age (4), and thus a large percentage of infants still require LT in the first 2 years of life (5,6).

BA patients have an excellent 5-year overall survival of 93% after KPE with or without subsequent LT (7), but early referral to a transplant center is essential, especially if cholestasis persists after KPE. However, the time taken to refer these children to a transplant center and/or to decide to list them for LT is currently too long and should be shortened (8). A paradigm shift in the intention to transplant is likely necessary, as late referral is associated with increased morbidity and mortality in affected patients. In this regard, centers with experience in comprehensive care of patients with BA starting in the neonatal period may achieve better outcomes.

A population-based study (9) showed that the second most common malformation after *in vitro* fertilization (IVF) was alimentary atresia (esophageal, small bowel, and anal atresia). In the report, 64 of 9111 patients presented associated malformations, and 3 cases had biliary tract anomalies that were not specified (BA, choledocal cysts). The association between IVF and BA was further described in only 2 case reports in the literature (10,11).

A historical perspective of pediatric LT at our center that included technical refinements over time and improvements in patient and graft survival rates was recently reported (12), based on our experience with 975 primary pediatric LT. The current report presents the clinical picture, patient characteristics, treatment strategies, and outcomes of BA patients with no previous treatment and were referred to us at an early age.

## Material and Methods

This is a retrospective cohort study to evaluate the outcomes of all patients who were referred to a single team, on an intent-to-treat basis, after presenting with BA with no previous treatment. This study was conducted at Hospital Sírio-Libanês and Hospital A.C. Camargo Cancer Center, in São Paulo, Brazil, from January 2001 to January 2021. All medical care for all patients, including diagnosis of BA, KPE, clinical follow-up, transplantation, and post-transplantation follow-up, was provided by the same pediatric hepatology and transplant team over the years. Thirty-nine patients were referred to our transplant center in this period and are the subject of this analysis. Two pediatric surgeons from the transplant team (JSN and PC) were responsible for the Kasai procedure in all cases and, when necessary, also for the LT. Data were acquired through retrospective review of medical records and from a prospectively collected database. The hospitals' ethics committees approved this study.

Technical aspects and outcomes in primary LT (12,13) and retransplantation (14) performed by our transplantation team were previously published and are not included in the present article.

### Patient assessment and management

All patients included in this study had BA. They were evaluated according to their age (days) at first assessment, clinical presentation (jaundice, choluria, alcoholic stools), physical examination (liver and spleen size, presence of ascites), laboratory values (liver function tests, infectious workup, serum protein electrophoresis), image studies (total abdominal ultrasound and hepatobiliary scintigraphy - DISIDA scan), liver biopsy, and intraoperative cholangiography when necessary. The patients were followed and divided into 3 groups: 1) Kasai-only group (K-only); 2) LT-only group (patients with portal hypertension - ascites and/or splenomegaly - who underwent liver transplantation); and 3) Kasai+LT (K+LT) group.

The indications for LT were: failure to obtain drainage from a Kasai operation within 3 months (chronic cholestasis), cholangitis leading to repeated hospitalizations, complicated portal hypertension (gastrointestinal bleeding or severe ascites that cannot be cured), uncorrectable severe coagulopathy, secondary hypoxia, or severe intractable pruritus impacting quality of life (8).

The transplant techniques, routine prophylaxis of vascular thrombosis, and post-transplantation clinical management have been described in detail by the authors in previous publications (12,15,16). There were no cases of incompatible ABO blood type transplant throughout the study period. All recipients were listed for deceased donor liver transplantation (DDT) in regional waiting lists; after 2006, the organ allocation organization in Brazil started following the Modified-PELD system (17). Although our transplant team has used living donor liver transplantation (LDLT) since 1995, all patients were listed for DDT. Which liver graft (DDT or LDLT) to use was decided based on availability from the moment patients were enrolled in the regional waiting lists.

Tacrolimus (FK 506, Prograf; Astellas Pharma Inc., Japan) and steroids were used for immunosuppression in the majority of the recipients.

### Statistical analysis and studied parameters

Means and medians were calculated to summarize continuous variables, and results were compared using Student's *t-*test or the Kruskal-Wallis test as the non-parametric test, when normal distributional assumptions were questionable. Categorical variables are reported as numbers and percentages. Differences between groups were assessed using chi-squared or Fisher's exact tests, when needed.

Patient and graft survival analysis was conducted according to the Kaplan-Meier product-limit estimates, and patient subgroups were compared using a two-sided log-rank test. All analyses were performed using the SPSS 21.0 statistical package (IBM, USA).

## Results

**Figure 1:**
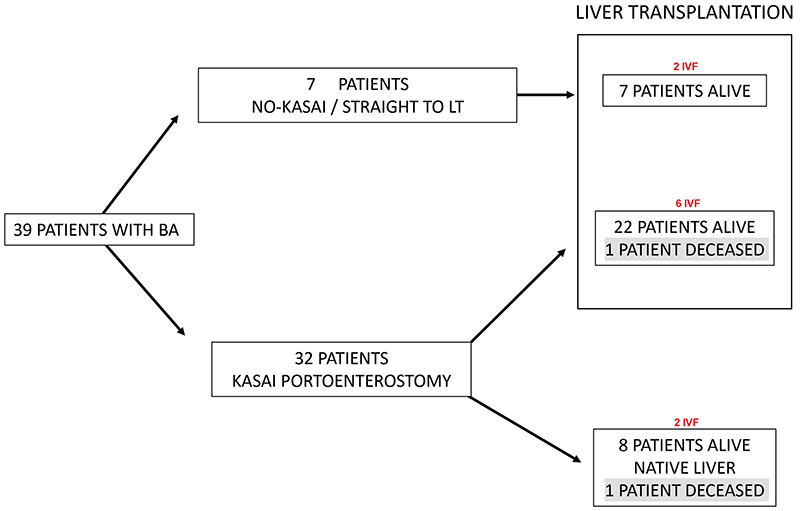
Treatment path for biliary atresia (BA) patients treated from an early age at a single center. LT: liver transplantation; IVF: *in vitro* fertilization.

**Figure 2:**
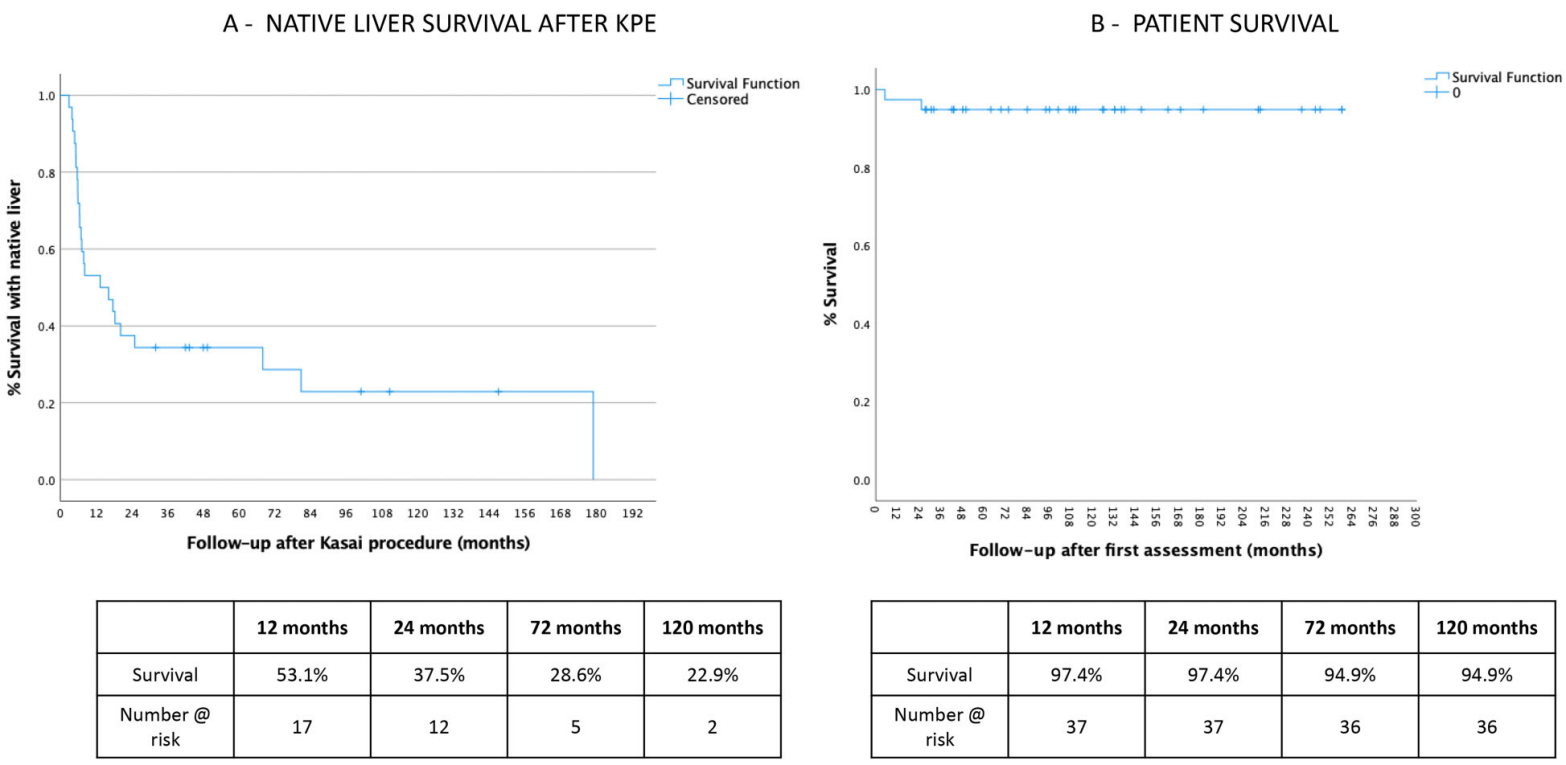
A, Kaplan-Meier survival probability of patients with native liver. **B**, Kaplan-Meier survival probability for all the studied cohort. KPE: Kasai-portoenterostomy.

Thirty-nine patients with BA were studied, and their treatment paths are illustrated in Figure 1. Seven patients did not undergo KPE and received liver grafts at various times (LT-only group). Thirty-two children underwent KPE; nine of them comprised the K-only group, 8 of which survived with their native liver a median time of 5 years (range 2 to 11) and 1 died during follow-up. Twenty-three patients were transplanted after KPE (K+LT group). Median follow-up in this group was 11 years (range 2 to 20), survival with native liver was 22.9% at 120 months of follow-up (Figure 2A), and overall patient survival was 94.8% at the same time point (Figure 2B).

In the K-only group (Supplementary Table S1), mean age at the procedure was 46.8±21.8 days, and mean body weight at KPE was 4.0±0.7 kg. Two patients (22%) in this group were conceived through IVF. One patient in the K-only group presented with ascites at diagnosis (abdominal ultrasound - patient 1) but underwent the procedure at 50 days old. Patient 2, with congenital heart disease (CHD) and biliary atresia splenic malformation (BASM) syndrome, died due to acute myocardial infarction 2 years after the KPE, with polycythemia secondary to severe CHD as a complication. All surviving patients in this group currently have normal bilirubin levels, but they all had episodes of cholangitis during follow-up.

The patients who underwent LT were only referred to our center with a mean age at diagnosis of 108.1±23.0 days (Supplementary Table S2). Two patients (28.5%) were conceived through IVF. All patients in the group presented with splenomegaly at diagnosis, and KPE was contraindicated. The mean Pediatric End-Stage Liver Disease (PELD) score at LT was 27.5±14.2, and the median time between diagnosis and transplantation was 168 days. They underwent LDLT with the left lateral segments (LLS), and one patient received a hyper-reduced LLS (Patient 6). All patients were alive with normal liver function at 6.9 years (range 1.5 to 12 years) of follow-up. Patients in this group presented the following complications: portal vein stenosis (Pts 1 and 3) and late portal vein (PV) thrombosis (Pt 7).

In the K+LT group, six patients (26%) were conceived through IVF (Tables 1 and 2). Table 1 focuses on the pre-Kasai status. The average age at KPE was 52.1±22 days (a non-significant difference from the age in the K-only group, P=0.4). Five patients presented with associated CHD and 1 patient with BASM syndrome; 3 patients presented with splenomegaly and one (15 days old) presented with both ascites and splenomegaly at diagnosis. There was a median time of 209 days between KPE and LT in this group (age at LT - age at KPE). The median age at LT in this group was 251 days. All patients except for one presented with splenomegaly before the transplant, 52% presented with cholangitis, and 4 patients had upper gastrointestinal bleeding prior to transplantation. The mean PELD score was 13.6±9.2. Patient 6 in this group died on the 20th day after receiving a split liver graft, due to pulmonary hypertension. The majority of liver grafts used were LLS (82.6%) from living donors, but there were also 2 left liver lobes. Post-transplantation complications in the K+LT group were: biliary stricture (Pts 3, 10, 13, and 22), biliary leak (Pt 12), late PV thrombosis (Pt 4), and PV stenosis (Pts 7 and 17).

**Table 1:** Characteristics of the Kasai+liver transplantation (LT) group; pre-Kasai data.

Pt	BW @Kasai (kg)	Age @Kasai (d)	Z-Score H/A @ Kasai	IVF	Comorbidities	Ascites @ diagnosis	Splenomegaly @ diagnosis	DB Pre-Kasai (mg/d)
1	4.3	37	-0.3	No	None	No	No	4.1
2	4.2	66	-1.5	No	BASM	No	No	8.1
3	3.9	23	-1.0	Yes	CHD	No	No	4.2
4	4.5	62	-1.7	No	None	No	No	4.0
5	3.7	48	-1.4	Yes	None	No	No	5.0
6	3.0	77	-3.0	No	None	No	No	8.6
7	3.0	46	-2.4	No	None	No	No	3.0
8	4.2	13	1.6	No	None	No	No	3.0
9	5.4	69	-0.2	No	None	No	Yes	3.9
10	3.9	41	-1.0	No	None	No	No	6.7
11	3.8	59	-2.0	Yes	None	No	No	4.7
12	3.6	40	-2.0	No	None	No	No	3.5
13	5.8	75	-1.0	No	CHD	No	No	3.1
14	4.6	82	-0.8	No	None	No	Yes	2.5
15	4.1	48	-0.6	No	None	No	No	3.9
16	3.9	77	-2.1	Yes	None	No	No	10.7
17	4.5	56	-1.0	Yes	CHD	No	No	6.0
18	3.6	60	-2.7	No	None	No	No	3.9
19	5.0	59	-0.2	No	CHD	No	No	3.6
20	4.3	15	-0.3	No	None	Yes	Yes	8.6
21	3.6	92	-4.6	Yes	CHD	No	No	6.3
22	2.6	20	-3.3	No	None	No	No	3.7
23	3.6	34	-1.6	No	None	No	No	7.6
Mean	4.0	52.1	-1.4	NA	NA	NA	NA	4.7
SD	0.7	22.0	1.2	NA	NA	NA	NA	2.2

Pt: patients; d: days; BW: body weight; H/A: height/age; DB: direct bilirubin, CHD: congenital heart disease; BASM: biliary atresia splenic malformation; IVF: *in vitro* fertilization; SD: standard deviation; NA: not available.

**Table 2:** Characteristics of the Kasai + liver transplantation (LT) group; pre- and post-LT data.

Pt	BW @LT (kg)	Age @LT (d)	Z-score H/A @ LT	DBPre-LT (mg/dL)	PELD	Spleen Pre-LT (cm)	Cholangitis Pre-LT	Upper GI Bleed Pre-LT	F-up (years)	DB @F-up (mg/dL)	Status
1	8.2	231	-0.5	6.4	23	4	Yes	No	19.7	0.4	Alive
2	6.9	275	-1.5	5.8	15	2	No	Yes	8.5	0.1	Alive
3	6.2	141	-1.5	15.3	12	3	No	No	1.5	0.2	Alive
4	7.2	206	-1.3	4.9	6	4	No	No	12.6	0.1	Alive
5	5.7	136	-1.8	10.5	17	5	No	No	16.8	0.1	Alive
6	6.2	204	-2.4	9.5	13	5	No	No	†	NA	Dead
7	6.1	222	-2.0	15.2	20	6	No	No	10.5	0.1	Alive
8	6.4	229	-2.3	9.2	25	4	Yes	No	20.7	0.1	Alive
9	50.0	5437	-1.5	0.8	11	12	Yes	No	18.8	0.2	Alive
10	6.9	198	-1.3	12.7	19	4	No	No	5.5	0.1	Alive
11	7.1	245	-1.1	14.6	19	7	Yes	No	9.7	0.1	Alive
12	5.5	276	-4.0	10.2	14	7	Yes	No	16.9	0.2	Alive
13	6.8	231	-1.9	13.8	15	5	No	Yes	20.4	0.2	Alive
14	6.3	633	-4.8	26.2	13	5	No	No	19.4	1.1	Alive
15	20.3	2473	-0.9	3.0	1	4	Yes	No	14.3	0.3	Alive
16	8.4	480	-1.3	4.6	8	6	Yes	No	7.4	0.3	Alive
17	6.3	251	-1.8	14.4	24	6	No	No	9.7	0.5	Alive
18	17.7	2100	0	9.5	13	6	Yes	No	10.3	0.1	Alive
19	10.5	546	0.2	1.4	3	6	Yes	Yes	3.0	1.4	Alive
20	7.6	260	-1.4	4.7	1	4	No	No	6.3	0.1	Alive
21	9.7	699	-1.8	4.3	1	6	Yes	Yes	7.0	0.2	Alive
22	4.5	192	-4.0	14.5	28	0	Yes	No	8.2	0.1	Alive
23	11.1	564	-0.4	6.8	15	7	Yes	No	2.0	1.7	Alive
Mean	10.0	705.0	-2.7	9.4	13.6	5.0	NA	NA	11.0	1.1	NA
SD	9.4	1189	4.8	6.0	9.2	2.3	NA	NA	6.2	2.1	NA

Pt: patients; BW: body weight; LT: liver transplantation; H/A: height/age; d: days; DB: direct bilirubin; PELD: pediatric end-stage liver disease; F-up: follow-up; GI: gastrointestinal; Spleen: cm from the left costal margin at physical examination; ^†^patient died 20 days after LT; NA: not available, SD: standard deviation.

Ten patients in this series (25.6%) were babies conceived through IVF, as shown in Supplementary Tables S1 and S2, and in Table 1. There was no difference in IVF incidence among the 3 studied groups (P=0.95). Four IVF patients (40%) presented with associated CHD *vs* 5 patients (17%) in the remaining groups (P=0.14). Two (20%) of the 10 patients were premature (gestational age <37 weeks) *vs* 1 of 29 (3.4%) in the non-IVF patients (P=0.09), and the median maternal age at birth was 35 years (range 33 to 41 years). None of the IVF patients presented other malformations of the alimentary tract. In regards to post-transplant survival, none of the IVF patients died during follow-up, and the post-transplant survival compared to the non-IVF patients was not statistically significant.

## Discussion

The available treatment strategies for BA have excellent long-term patient survival, as shown in the present study. A particular characteristic of this group was that children were referred to a center that provided care from an early age and all treatment options were timely offered. Likewise, Ramos-Gonzalez et al. (7) showed a long-term survival of 93% in a cohort of 81 BA patients who were treated from an early age. The 10-year transplant-free survival was 36%; 36 patients (44%) ultimately required transplantation. In their report, there is no mention of patients who underwent LT only, and in 5 patients, KPE was performed after 90 days of age. In the majority of centers, however, the diagnosis of BA and KPE are under the responsibility of pediatric surgeons and pediatricians. Patients with persistent cholestasis and/or portal hypertension are then referred to LT centers, where transplant surgeons and pediatric hepatologists are in charge of LT and follow-up. This transition of care may be one of the reasons many patients are referred late for liver transplantation.

Primary liver transplantation for patients with BA may be required in 3 to 16% of the cases (18,19), typically in infants with late presentation (after 100 days) and consequent overt cirrhosis. LeeVan et al. (20), in a cohort of 626 patients, compared the outcomes of patients who received primary LT (313 patients) and those who had KPE before LT (n=313), and showed that patients who underwent primary LT had a markedly reduced risk of long-term mortality compared with patients initially managed with KPE. Conversely, in a cohort of 347 primary pediatric LT for BA (15), patients with a previous KPE who were transplanted more than 1-year post-KPE (115 patients - Kasai late failure) presented better patient and graft survival than patients who received a primary LT (138 patients) and KPE early failure (KPE to LT <1 year, 94 patients). In the present study, 18% of the patients underwent primary LT due to portal hypertension, even though 2 of 7 patients were under 90 days old at diagnosis. All patients in this group presented cholestasis with cirrhosis and portal hypertension.

Survival with the native liver after KPE was 22.9% in the present series, where the median age at KPE was 46.8 days. The key outcome measure for the success of KPE is the percentage of patients in whom jaundice regresses arbitrarily to normal values (18). Despite normal bilirubin levels, all patients in the Kasai-only group presented cholangitis at a median follow-up of 5 years, and 50% of the surviving patients presented splenomegaly at physical examination, which is an indicator of portal hypertension. In the series of Davenport et al. (18) survival with a native liver at 5 years was 46%, with patient survival estimates of 90% at the same time point. Venkat et al. (21), in an effort to determine risk factors for disease progression in patients who had survived with native liver at 2 years of age, showed that the cumulative incidence of either LT or death or occurrence of sentinel events (SE) was 23.7 and 21.5%, respectively. SE was considered as new onset ascites, hepatopulmonary syndrome (HPS), or gastrointestinal (GI) bleed; however, cholangitis after KPE was not included as an SE in the study.

For most patients, KPE is only a temporary solution and a bridge to liver transplantation (7). Indeed, Fanna et al. (22) recently published an experience in France with 1428 BA children (30 years of follow-up, 1986 to 2015). The actual BA patient survival (from diagnosis) was 81% and included results from many centers. Patients from the most recent cohort in that study (2010 to 2015) had 87% 5-year survival, an improvement from 72% in the first part of the study (1986 to 1996). This trend is naturally expected with accumulated experience over the years, and the awareness that the 60-day paradigm for KPE must be shifted to early intervention in the course of the disease (23).

The association of IVF with BA was an unexpected finding in our series (25.6%). Particular to the cohort described herein, most of the patients treated from an early age at our center were from families with better economic resources and access to fertilization treatment. Two reports in the literature previously described such association (10,11). In the first (10), a late-preterm (gestational age 36 weeks, birth body weight 1,896 g) female baby, after correction of a midgut volvulus that required bowel resection, presented with biliary atresia and underwent KPE at 52 days of life. In the second (11), a premature infant (32 weeks, 1,509 g) diagnosed with both duodenal atresia and BA underwent a successful duodenoduodenostomy procedure with bypass of the duodenal web at 2 days of life, and subsequently a KPE at 39 days of life. The authors credited the combined malformations encountered to prematurity rather than IVF, since although IVF does appear to have an increased risk for some congenital anomalies, there is not a significant increase in anomalies of the gastrointestinal tract (24). Conversely, Ericson and Källén (9) showed that the second most common malformation after IVF were alimentary atresias. These might be associated with the same type of disturbance that increases the risk for monozygotic twinning and could be a direct effect of the IVF procedure.

The incidence of CHD in patients with BA has been reported to be as high as 15% (25), and the historical cohort from our group showed similar rates (16.6%, 325 BA, 54 patients with CHD) (26). Even though no statistical difference was found, 40% of BA+IVF patients had CHD. According to Källén et al. (24), when the malformations are divided into subgroups, some appear to be more strongly associated with IVF, such as central nervous system malformations (including neural tube defects), cardiovascular defects, limb reduction defects, and congenital malformation syndromes.

With the available treatment strategies, excellent patient survival is expected for patients with BA. In the present study, overall patient survival was 94.8%. All patients who survived with native liver (22.9%), despite normal bilirubin levels, presented with cholangitis during follow-up. In the long term, post-transplant survival was 96.6%.

Because of the small number of patients in this cohort it is not possible to establish the real impact of IVF in the clinical outcome of patients with BA. Also, we can only speculate whether IVF increases the risk of developing BA. The literature is limited, and there are only two case reports describing this occurrence (10,11), as mentioned before. Therefore, further studies are required to better understand the possible link between IVF and BA.

## Supplementary Material

Click to view [pdf].
